# Identification of surfactins and iturins produced by potent fungal antagonist, *Bacillus subtilis* K1 isolated from aerial roots of banyan (*Ficus benghalensis*) tree using mass spectrometry

**DOI:** 10.1007/s13205-013-0151-3

**Published:** 2013-07-04

**Authors:** Khyati V. Pathak, Hareshkumar Keharia

**Affiliations:** BRD School of Biosciences, Sardar Patel University, Sardar Patel Maidan, Satellite Campus, Vadtal Road, P.O. Box 39, Vallabh Vidyangar, 388120 Gujarat India

**Keywords:** Endophyte, *B. subtilis* K1, Surfactin, Iturin, Mass spectrometry

## Abstract

The banyan endophyte, *Bacillus subtilis* K1, produces a complex mixture of lipopeptides exhibiting potent antifungal activity. These lipopeptides were purified by high-performance liquid chromatography and analyzed using MALDI-TOF-MS as well as liquid chromatography coupled with ESI-MS. A heterogenous mixture of lipopeptides belonging to three different families of cyclic lipopeptides, viz., fengycins, iturins and surfactins, was detected in the cell-free extracellular extract of *B. subtilis* K1. The detailed mass spectrometric characterization revealed the presence of four variants of iturin A and three variants of iturin C varying in the β-amino fatty acid chain length from C_13_ to C_17_. The MS/MS of monovalent alkali metal ion adducts (Na and K) of iturin suggested the Glu^4^ as a binding site for metal ion. The LC-ESI-MS/MS analysis of surfactins enabled the identification of seven surfactin variants with the variations in Val/Ile/Leu at position 4 and C_13_–C_17_ β-hydroxy fatty acids. This study demonstrates the application of tandem mass spectrometry in identification of closely related lipopeptides from a heterogenous mixture obtained from a natural source. Furthermore, this is the first report of an endophytic bacillus strain co-producing so many variants of surfactins and iturins.

**Electronic supplementary material:**

The online version of this article (doi:10.1007/s13205-013-0151-3) contains supplementary material, which is available to authorized users.

## Introduction

Endophytes are microorganisms which reside within plant tissues without causing any harm to host and they represent potential source of novel bioactive metabolites (Strobel 2006). In search for endophytes with potential biocontrol activity, we isolated bacilli strains exhibiting potent antifungal activity from hanging roots of Banyan tree. The *B. subtilis* K1, isolated from the aerial roots of banyan tree, was found to inhibit growth of *Aspergillus niger*, *Alternaria brunsii*, *Fusarium oxysporum*, *Cladosporium herbarum* 1112, *Candida albicans* and *Lasiodiplodia theobromae* ABFK1 (Pathak et al. 2012; Pathak and Keharia 2013). The mass spectrometric analysis of crude lipopeptide extract from cell-free culture supernatant of *B. subtilis* K1 revealed that it was a complex mixture of iturins, surfactins and fengycins. This prompted us to characterize these cyclic lipopeptides employing tandem mass spectrometry.

Bacilli in general are known for their ability to produce wide array of broad spectrum antibiotics synthesized either ribosomally or non-ribosomally (Katz and Demain 1977; Stein 2005; Ongena and Jacques 2007). Among these, the non-ribosomally synthesized lipopeptide antibiotics are well documented for their antifungal, antibacterial, antiviral, insecticidal, antitumor and surfactant activities (Winkelmann et al. 1983; Vanittnakom et al. 1986; Vollenbroich et al. 1997; Kracht et al. 1999; Stein 2005; Ongena and Jacques 2007; Kim et al. 2007, 2010; Geetha et al. 2010). The production of surfactins, iturins and fengycins by various strains of *B. subtilis* has been reported by several researchers (Winkelmann et al. 1983; Vanittnakom et al. 1986; Vollenbroich et al. 1997; Kracht et al. 1999; Kim et al. 2007; Nagorska et al. 2007; Ongena and Jacques 2007; Kim et al. 2010; Geetha et al. 2010). The lipopeptides belonging surfactin family are β-hydoxy hepta cyclic depsipeptides with possibilities of Ala, Val, Leu or Ile amino acid variations at positions 2, 4, and 7 in cyclic depsipeptide moiety and C_13_ to C_16_ variation in β-hydroxy fatty acid chains (Peypoux et al. 1994; Kowall et al. 1998; Hue et al. 2001). Surfactin is well known for its extraordinary surfactant activity and has also been demonstrated to possess antiviral, antitumor and insecticidal activities (Ongena and Jacques 2007, Vollenbroich et al. 1997; Kracht et al. 1999; Kim et al. 2010; Geetha et al. 2010). Iturins are broad spectrum potent antifungal heptapeptides cyclized by amide bond formed between α-COO group of seventh amino acid and β-NH_2_ group of β-amino fatty acid (βAA), which in turn is peptide bonded through its α-COO group to N-terminal amino acid. The members of iturin family exhibit heterogeneity at 1, 4, 5, 6 and 7 amino acid position/s in the peptide moiety as well as in the βAA length, which varies from 14 to 17 carbons. On the basis of variation of amino acids in peptide moiety, iturins have been classified as: iturin A, iturin C, iturin D, iturin E, bacillomycin D, bacillomycin F, bacillomycin L, bacillomycin Lc and mycosubtilin (Winkelmann et al. 1983; Peypoux et al. 1981, 1984, 1986; Isogai et al. 1982; Gong et al. 2006; Romero et al. 2007; Pecci et al. 2010). Among all the iturins, iturin A has been found to be most potent antifungal lipopeptide and is secreted by most bacilli strains exhibiting strong as well as broad spectrum antifungal activity (Romero et al. 2007; Pecci et al. 2010). Different strains of bacilli exhibit diversity in production of cyclic lipopeptides, with most strains reported to produce lipopeptides belonging to only one family while few reported to be co-producers of lipopeptides belonging to two or all three families (Vater et al. 2002; Nagorska et al. 2007; Romero et al. 2007; Pecci et al. 2010). Furthermore, the fungal antagonistic activity of *Bacillus* sp. has been correlated with the amount as well heterogeneity in the production of lipopeptides (Nagorska et al. 2007; Ongena and Jacques 2007; Pecci et al. 2010).

The present study describes detailed mass spectrometric analysis of the surfactins and iturins produced by *B. subtilis* K1. To the best of our knowledge, this is a first report on mass spectrometric characterization of such a heterogenous mixture of surfactin and iturin variants co-produced by a single bacillus strain.

## Materials and methods

### Bacterial strain and production of lipopeptides

The *B. subtilis* K1 exhibiting broad spectrum antifungal activity was isolated from the aerial roots of banyan tree (Pathak et al. 2012). For production of lipopeptides, an inoculum was prepared by inoculating a single colony of *B. subtilis* K1 into 50 mL of Luria broth in 250-mL Erlenmeyer flask and incubating at 30 °C for 12 h. The 12-h-old inoculum was used to seed 250 mL of glucose yeast extract (GY medium: composition in g/L: glucose, 10.00; yeast extract, 1.00; KH_2_PO_4_·2H_2_O, 1.00; K_2_HPO_4_·2H_2_O, 1.00; MgSO_4_·7H_2_O, 0.20; CaCl_2_·2H_2_O, 0.02; FeSO_4_·2H_2_O, 0.05) medium in 1,000-mL flask to an initial OD_(600 nm)_ of ~0.05 and incubated at 30 °C for 72 h on orbital shaker (150 rpm).

### Extraction and purification of lipopeptides

The 72-h-old fermentation broth of *B. subtilis* K1 was centrifuged at ~10,000×*g* at 4 °C for 20 min. The supernatant was collected and its pH was lowered to pH 2 using 6 N HCl and incubated at 4 °C for 4 h in order to precipitate lipopeptides. The acid precipitates were recovered by centrifugation at ~10,000×*g* at 4 °C for 20 min, and pellet thus obtained was solubilized in anhydrous methanol (Pathak et al. 2012). The methanolic lipopeptide extract was further concentrated using vacuum evaporator (Buchi Switzerland).

The lipopeptides were purified by HPLC employing a semi-preparative reverse phase C-18 HPLC column (4.6 × 250 mm, 10 m particle size, 90 pore size) using methanol/water/0.1 % trifluroacetic acid solvent system. The elution of lipopeptides was done using a gradient of 80–95 % methanol (v/v) for 50 min, 95 % methanol (v/v) for 5 min and 95–80 % for 5 min (Pathak et al. 2012). The lipopeptide fractions were then analyzed by mass spectrometry.

### Mass spectrometry of lipopeptides

#### MALDI-TOF mass spectrometry (MALDI-TOF-MS)

All the lipopeptide fractions obtained by reverse phase HPLC were subjected to MALDI-TOF-MS analysis using Ultraflex TOF/TOF spectrometer (Bruker Daltonics, Billericia, MA, USA and Bremen, Germany). The lipopeptide fractions were mixed with equal volume of α-cyano-4-hydroxy-cinnamic acid matrix and spot applied on the target plate. The mass spectrometric (MS and MS/MS) data acquisition was carried out as per the method described previously (Pathak et al. 2012).

#### Liquid chromatography electrospray ionization MS (LC–ESI–MS)

LC–ESI–MS spectra were acquired over the mass range of *m/z* 50–2,000 by passing the analyte through a Zorbax 300SB-C18 column (Santa Clara, CA, USA) (4.6 × 150 mm, 5 μm particle size) using MeOH/H_2_O/0.1 % (v/v) formic acid on an Agilent 1100 HPLC system, coupled with HCT Ultra ETD II (Bruker Daltonics, Bremen, Germany) ion trap mass spectrometer in positive ion mode. A gradient (80–95 % MeOH in 50 min, 95 % MeOH for 5 min and 95–80 % MeOH in 5 min) pumped at a constant flow rate of 0.2 mL/min was used with a run time of 60 min. The data were acquired in an auto MS^2^ mode using collision-induced dissociation method for fragmentation and data were analyzed by Data Analysis software ver. 4.0 (Bruker Daltonics, Bremen, Germany).

#### Minimum inhibitory concentration (MIC) of purified lipopeptides

Stock solutions (1 mg/mL) of iturin A2, iturin A2/A3/A4 and fengycin were prepared by dissolving lyophilized fractions in MeOH and analyzed for activity against *A. niger* 40211, *A. flavus*, *A. parasiticus*, *F. oxysporum* 1072, *Chrysosporium indicum*, *Candida albicans*, *Trichosporon* sp. 1110, *Alternaria brunsii* (2), and *Cladosporium herbarum* 1112. Fungal spore suspension was prepared by harvesting spores into sterile distilled water and the spore counts were determined using haemocytometer. In case of *Candida albicans* and *Trichosporon* sp. 1110, culture suspension was prepared by cultivating them in 50-mL potato dextrose broth under agitated condition (150 rpm) at 30 °C for 10–12 h. The cell numbers were determined using a hemocytometer and adjusted to 1 × 10^6^ cells/mL by appropriate dilution with sterile distilled water. The MIC of pure lipopeptides were determined by double dilution technique against susceptible fungal cultures in sterile 96-well microtiter plates with each well containing 100 μL of potato dextrose broth. After dilution ~10^2^ spores of test fungus were inoculated into each well. To control wells, corresponding aliquot of MeOH instead of sample was added. The plates were incubated for 24–48 h at 30 °C and MIC values for each fraction were determined against susceptible test fungi based on the highest dilution showing no growth.

## Results and discussion

The MALDI-TOF MS analysis of HPLC purified fractions of crude antifungal extract obtained from cell-free supernatant of *B. subtilis* K1 revealed the presence of compounds with molecular mass ions in the range, *m/z* 1,028–1,109, *m/z* 994–1,065 and *m/z* 1,421–1,566, which were assigned as iturins, surfactins and fengycins, respectively (Kowall et al. 1998; Vater et al. 2002; Williams and Brodbelt 2004; Gong et al. 2006; Romero et al. 2007; Pathak et al. 2012). The characterization of iturins and surfactins produced by *B. subtilis* K1 is described below:

### Identification and characterization of iturins

**Fig. 1:**
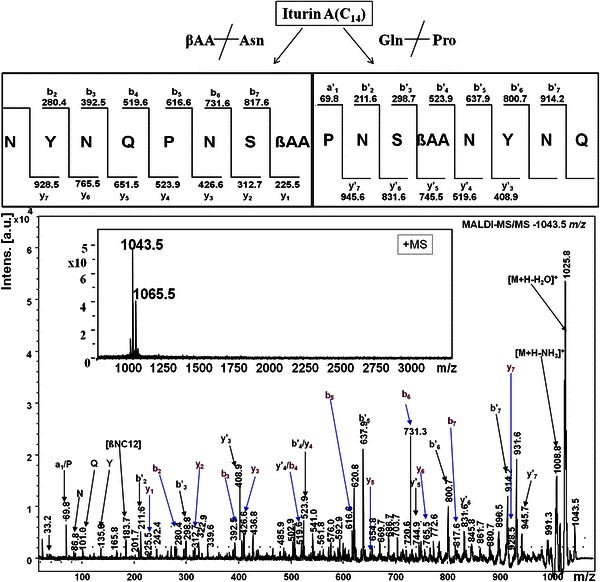
MALDI-MS/MS spectrum of [M+H]^+^ ion at *m/z* 1,043.5 from HPLC fraction P3

The iturins containing fraction with *m/z* range 1,028–1,109 eluted in HPLC peak no. P2–P6 and were further analyzed by MALDI-TOF MS/MS for peptide sequence determination. The MALDI-TOF MS of HPLC peaks P2–P6 corresponded the molecules with *m/z* 1,029.5 (P2), 1,043.5 (P3), 1,057.5 (P4), 1,071.5 (P5) and 1,072.5 (P6) (for HPLC chromatogram see publication Pathak et al. 2012). The molecules at *m/z* 1,029.5, 1,043.5, 1,057.5 and 1,071.5 differed in their masses by 14 or multiples of 14 Da while the *m/z* 1,072.5 differed from 1,071.5 by 1 Da, suggesting them to be members of a same family (Gong et al. 2006; Williams and Brodbelt 2004; Pecci et al. 2010). Two additional sets of *m/z* ions (with corresponding mass difference of 22 and 39 Da) were also detected which were putatively assigned as sodium adducts (*m/z* 1,051.5, 1,065.6, 1,079.5 and 1,093.5) and potassium adducts (*m/z* 1,067.5, 1081.5, 1,095.5 and 1,109.5) of corresponding iturins (Vater et al. 2002; Gong et al. 2006; Romero et al. 2007; Pecci et al. 2010). The appearance of sodium and potassium adducts along with their protonated species is a common feature in mass spectral studies of peptides under conventional MALDI as well as electron spray conditions (Eckart 1994; Hue et al. 2001; Rutenbach et al. 2001; Vater et al. 2002). The MS/MS of the cyclic peptides results into cleavage of either peptide bond or ester bond in the peptide backbone after gas phase protonation, which leads to formation of the protonated ring-opened forms with different linear sequences (Eckart 1994; Hue et al. 2001). In MALDI-MS/MS spectrum of protonated precursor ion at *m/z* 1,043.5, series of *a*-, *b*-, *b′*-, *y*-, *y′*-ions, immonium ions, ions with loss of H_2_O or NH_3_ or H_2_O + NH_3_ and internal fragment ions could be assigned (Fig. 1). These observed product ions were derived from two distinct modes of cleavage of the cyclopeptide ring, corresponding to initial fragmentation at the β-amino acid (βAA)-Asn site and Gln-Pro peptide bonds. The MS/MS spectrum of other protonated precursor ions at *m/z* 1,028.5, 1,043.5, 1,071.5 also yielded similar profile of product ions. The general sequence derived from the fragment ions formed upon cleavage at C-terminal end of βAA in MS/MS spectra of *m/z* 1,028.5, 1,043.5, 1,071.5 was N-Y–N-Q-P-N-S-βAA whereas, the sequence deduced from Pro^5^ directed cleavage was P-N-S-βAA-N-Y-N-Q. A third series fragment ions at *m/z* 225.5 (βAA), *m/z* 339.6 (βAA-N), *m/z* 502.9 (βAA-N-Y-), *m/z* 616.6 (βAA-N-Y-N), *m/z* 744.9 (βAA-N-Y-N-Q) and *m/z* 201.7 (S-N), *m/z* 298.8 (S–N-P), *m/z* 426.6 (S-N-P-Q), *m/z* 541.0 (S-N-P-Q-N), *m/z* 703.7 (S-N-P-Q-N-Y-), *m/z* 817.6 (S-N-P-Q-N-Y-N) were found in the MS_2_ spectrum of *m/z* 1,043.5. This fragment series suggested yet another ring cleavage site, i.e. amide bond between βAA and Ser^1^ of iturin homolog. The ion at *m/z* 183.7 seemed to have formed due to loss of 41 Da (–CH_2_–C=O)^+^ from βNC_14_ and was assigned as βNC_12._ The lipopeptide sequences derived from the MS/MS data for the molecules at *m/z* 1,029.5, 1,043.5, 1,057.5 and 1,071.5 were assigned as iturin A homologs, based on published literature (Vater et al. 2002; Isogai et al. 1982; Gong et al. 2006; Romero et al. 2007). The Pro-directed cleavage has been reported by Vater et al. (2002) and Gong et al. (2006). The cleavage of peptidyl-prolyl bond is ubiquitously observed in the tandem mass spectra of peptide containing proline (Papayannopoulos 1995; Rutenbach et al. 2001; Vater et al. 2002; Sabareesh et al. 2007). The basic nature of tertiary amide nitrogen of proline in its N-terminal peptide bond makes it susceptible towards low-energy fission and therefore it readily undergoes dissociation under gas phase (Papayannopoulos 1995; Sabareesh et al. 2007).

Gong et al. (2006) reported the ring opening in iturins due to cleavage of peptide bond between Tyr^2^ and Asn^1^ while, Vater et al. (2002) reported the ring opening due to cleavage of amide bond between βAA and Ser^7^. In our studies, we observed ring opening due to cleavages of amide bonds between βAA and Asn^1^, βAA and Ser^7^ as well as Gln^4^ and Pro^5^. The variation in pattern of ring opening in iturin A homologues could be due to different methods employed for their characterization. Gong et al. (2006) used FAB-TOF/TOF and Vater et al. (2002) used PSD-MALDI, whereas we studied fragmentation of purified iturin A homologues using MALDI-LIFT. The MS/MS spectra of all the three protonated species of iturin showed the low-mass ion region containing immonium ions as well as internal fragment ions. The immonium ions of amino acids, Q (*m/z* 101.4), Y (*m/z* 136.5) and P (*m/z* 70.3) could be identified in MS/MS spectra of all these three iturins while the ion at *m/z* 339.7 could be assigned as an internal ion of tripeptide −NPQ (Fig. 1). The presence of immonium ions along with internal ions have also been reported by Gong et al. (2006).

In the *b*- and *y*-type fragmentation of *m/z* 1,029.5, 1,043.5 and 1,057.5 and 1071.5, *y*-ion series of all four [M+H]^+^ precursor ions differed by 14 Da, whereas in case of*b*′- and*y*′-type fragmentation, *b*_*1*_*′b*-_*3*_*′* ions remained same while *b*_*4*_^*′*^*b*-_*7*_*′* ions differed from each other by 14 Da. The 14 Da difference observed in both cases suggested that the metabolites with *m/z* 1,029.5, 1,043.5 and 1,057.5 and 1,071.5 differed only in carbon length of βAA and thus were assigned as iturin A homologues with 13-, 14- and 15- and 16-carbon βAA, respectively (Table 1). Iturin A homologues have been classified into eight different groups (iturin A_1_–A_8_) based on the heterogeneity of βAA (chain length as well as isoforms of βAA viz., normal, iso and anteiso) (Isogai et al. 1982; Gong et al. 2006). In present study, *B. subtilis* K1 was found to produce a mixture of iturins consisting of at least three homologues of iturin A, of which *m/z* 1,029.5, *m/z* 1,043.5 and *m/z* 1,071.5 could be assigned as iturins A1, A2 and A6, respectively. The iturins A3, A4 and A5 are isomers (normal, iso and anteiso with respect to βAA) with same molecular mass of 1,057.5 and thus, *m/z* 1,057.5 may be one of these three iturin A isomers, which cannot be distinguished by mass spectrometry. Along with these four protonated iturin A species, fifth species with *m/z* 1,072.5 of iturin was also detected. The molecular mass ion at *m/z* 1,072.5 differed from 1,071.5 by 1 Da and it is difficult to separate the species varying by 1 Da in MS spectrum. Moreover, in MALDI-MS/MS spectrum of *m/z* 1,072.5 most of the fragmentation belonging to iturin A species was at *m/z* 1,071.5 therefore, to determine the identity of iturin species at *m/z* 1,072.5, LC-ESI-MS/MS method was used. In the ESI-MS spectrum of molecular mass ions ranging within the *m/z* 1,040 to *m/z* 1,080 (eluted within the time range of 9–23 RT), three isotopic peak clusters with mono isotopic masses at *m/z* 1,043.5, 1,057.5, 1,072.5 were observed (Fig. S1). The isotopic distribution in these peak clusters seemed to be abnormal as the intensity of second isotopic peak (1,044.5, 1,058.5 and 1,072.5) in each cluster is higher than the first isotopic peak. Moreover, these compounds are falling in the *m/z* range where normal isotopic distribution is anticipated. This type of isotopic distribution in the fengycins has been previously described and has been attributed to the presence of two fengycins differing by 1 Da in same isotopic cluster (Pathak et al. 2012). Thus, the presence of iturins differing by 1 Da may be attributed to the abnormal distribution in the iturin isotopic clusters observed in present study. According to literature, iturin A and iturin C are the homologues varying from each other by 1 Da due to variation of Asn to Asp at amino acid position 1 (Park et al. 1995). To confirm 1-Da variation in the peptide sequences, each of these ions was selected for MS–MS analysis. Similarly, the sequences P-N-S-βAA-D-N-Q and Q-P-N-S-βAA-D-Y-N were deduced from the MS/MS analysis of *m/z* 1,058.5 (Fig. S2). The MS/MS spectra of ions at *m/z* 1044.5, 1,072.5 also yielded similar peptide sequences with variation of βAA fatty acids chain length. Using LC-MS/MS approach, molecular mass ions at *m/z* 1,044.5, 1,058.5 and 1,072.5 were identified as iturin C homologues with C14 to C16 βAA fatty acids (Park et al. 1995). To the best of our knowledge, this is the first report on the identification of iturin A and iturin C (differing by 1 Da) co-produced by a *B. subtilis* strain, using LC-ESI-MS/MS.

**Table 1:** Iturin homologues secreted by *B. subtilis* K1

Mass (*m/z*)	Peptide sequence	Identification of iturins	References
1,029.5	Cyclo(βAA-N-Y-N-Q-P-N-S)	(C_13_) Iturin A1[M+H]^+^	This work (Gong et al. 2006)
1,051.5		(C_13_) Iturin A1[M+Na]^+^	
1,067.5		(C_13_) Iturin A1[M+K]^+^	
1,043.5	Cyclo(βAA-N-Y-N-Q-P-N-S)	(C_14_) Iturin A2[M+H]^+^	This work (Gong et al. 2006; Romero et al. 2007)
1,065.5		(C_14_) Iturin A2[M+Na]^+^	
1,081.5		(C_14_) Iturin A2[M+K]^+^	
1,057.5	Cyclo(βAA-N-Y-N-Q-P-N-S)	(C_15_) Iturin A3/A4/A5[M+H]^+^	This work (Romero et al. 2007)
1,079.5		(C_15_) Iturin A3/A4/A5[M+Na]^+^	
1,095.5		(C_15_) Iturin A3/A4/A5[M+K]^+^	
1,071.5	Cyclo(βAA-N-Y-N-Q-P-N-S)	(C_16_) Iturin A6/A7[M+H]^+^	This work (Romero et al. 2007)
1,093.5		(C_16_) Iturin A6/A7[M+Na]^+^	
1,109.5		(C_16_) Iturin A6/A7[M+K]^+^	
1,044.5	Cyclo(βAA-D-Y-N-Q-P-N-S)	(C_14_) Iturin C1[M+H]^+^	This work (Williams and Brodbelt 2004)
1,058.5	Cyclo(βAA-D-Y-N-Q-P-N-S)	(C_15_) Iturin C2[M+H]^+^	This work (Williams and Brodbelt 2004)
1,072.4	Cyclo(βAA-D-Y-N-Q-P-N-S)	(C16) Iutrin C3[M+H]^+^	This work (Williams and Brodbelt 2004)

*βAA* β-amino acid

**Fig. 2:**
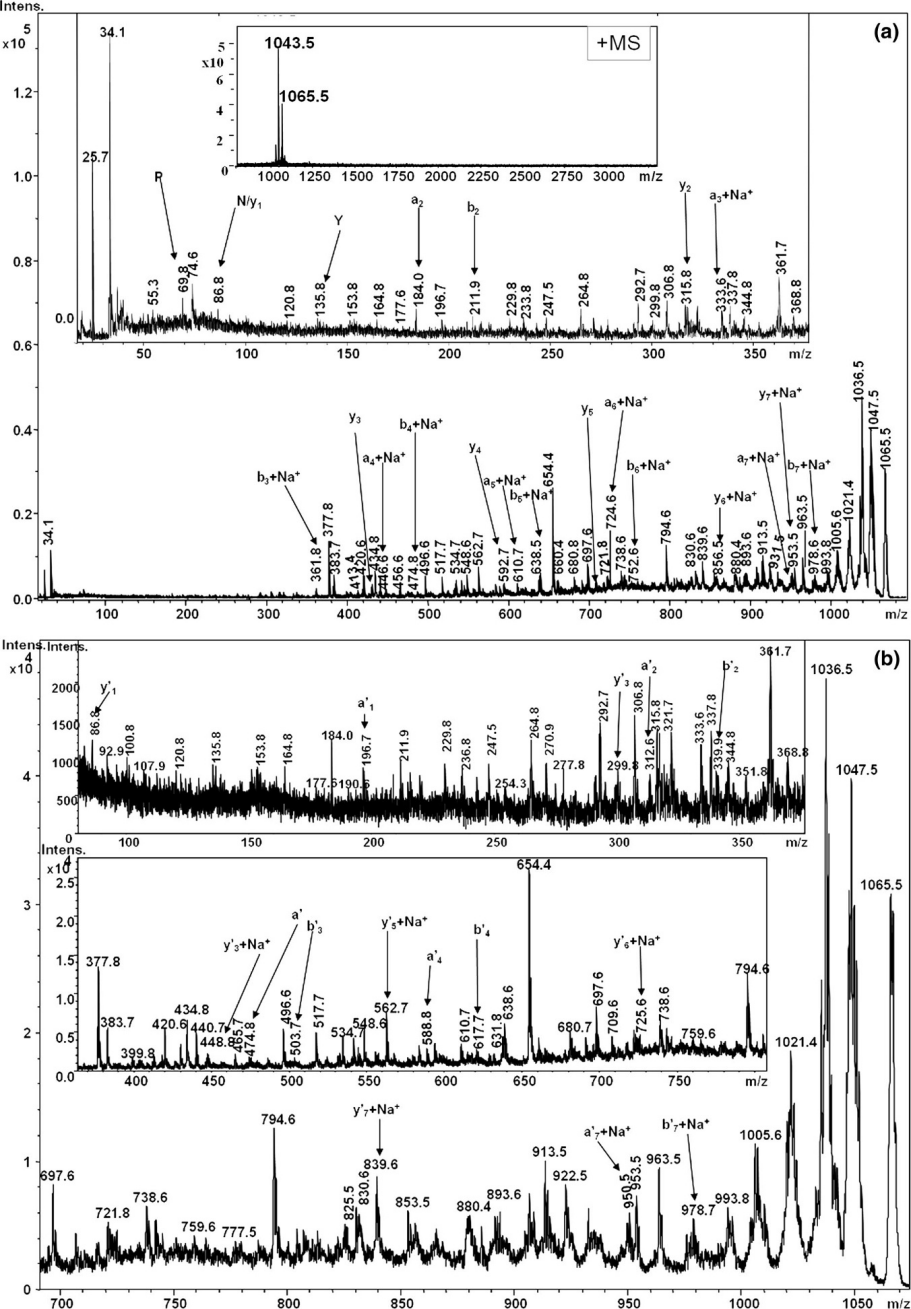
MALDI-MS/MS spectrum of [M+Na]^+^ ion at *m/z* 1,065.5 from HPLC fraction P3. The product ions, *a*-, *a* + Na-, *b*-, *b* + Na-, *x*-, *x* + Na-, *y*-, *y* + Na- assigned in (**a**) represents peptide sequence upon gas phase ring opening due to peptide bond cleavage between Ser_1_-Asn_2_ while the product ions, *a*′-, *a*′ + Na-, *b*′-, *b*′ + Na-, *x*′-, *x*′ + Na-, *y*′-*, y*′ + Na- and assigned in (**b**) shows peptide sequence upon ring opening between Ser^1^-βAA

**Fig. 3:**
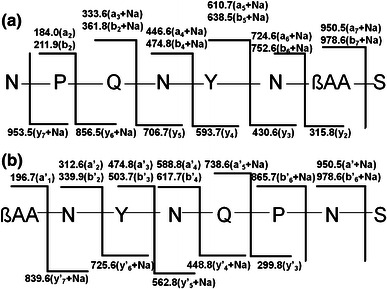
Peptide sequence assignments of [M+Na]^+^ ion at *m/z* 1,065.5

The precursor ions at *m/z* 1,051.5, 1,065.5, 1,079.5 and 1,093.5 with mass difference of 22 Da from ions at *m/z* 1,029.5 and 1,043.5, 1,057.5 and 1,071.5 were assigned as sodium adducts of corresponding iturin homologues. Each of these sodium adducts of iturin were subjected to MALDI-TOF MS/MS analysis and the fragment ions were analyzed. In MALDI-TOF MS/MS of [M+Na]^+^ at *m/z* 1,065.5, *a*-, *a′*-, *b*-, *b′*-, *y*- and *y′*-fragment ions could be assigned (Fig. 2). The fragment ions at *m/z* 1,021.4, 1,036.8 and 1,047.8 observed in MS/MS spectrum of [M+Na]^+^ ion at *m/z* 1,065.5 could be assigned as product ions formed upon loss of –CONH_2_, –C=O and NH_3_, respectively from the parent ion. The ions observed at *m/z* 794.6 [CH_2_-CO-N-Y-N-Q-P-N + Na]^+^, 839.6 [S-N-P-Q-N-Y-N +Na]^+^ and 913.5 [N-P-Q-N-Y-N- βNC13 + Na]^+^ were assigned as internal fragment ions. The sequences deduced from the fragmentation patterns of four sodium adducts of iturin A were N-P-Q-N-Y-N-βAA-S (Fig. 3a) and βAA-N-Y–N-Q-P–N-S (Fig. 3b), respectively. All the *b*-ions corresponding to both sodium adducts (*m/z* 1,065.5 and 1,079.5) were found to be same except *b*_*6*_ and *b*_*7*_ ion. The *b*_*6*_ and *b*_*7*_ ions of *m/z* 1,065.0 and 1,079.7 varied from each other by 14 Da, which may be attributed to the difference in the chain length of βAA by single –CH_2_.

**Fig. 4:**
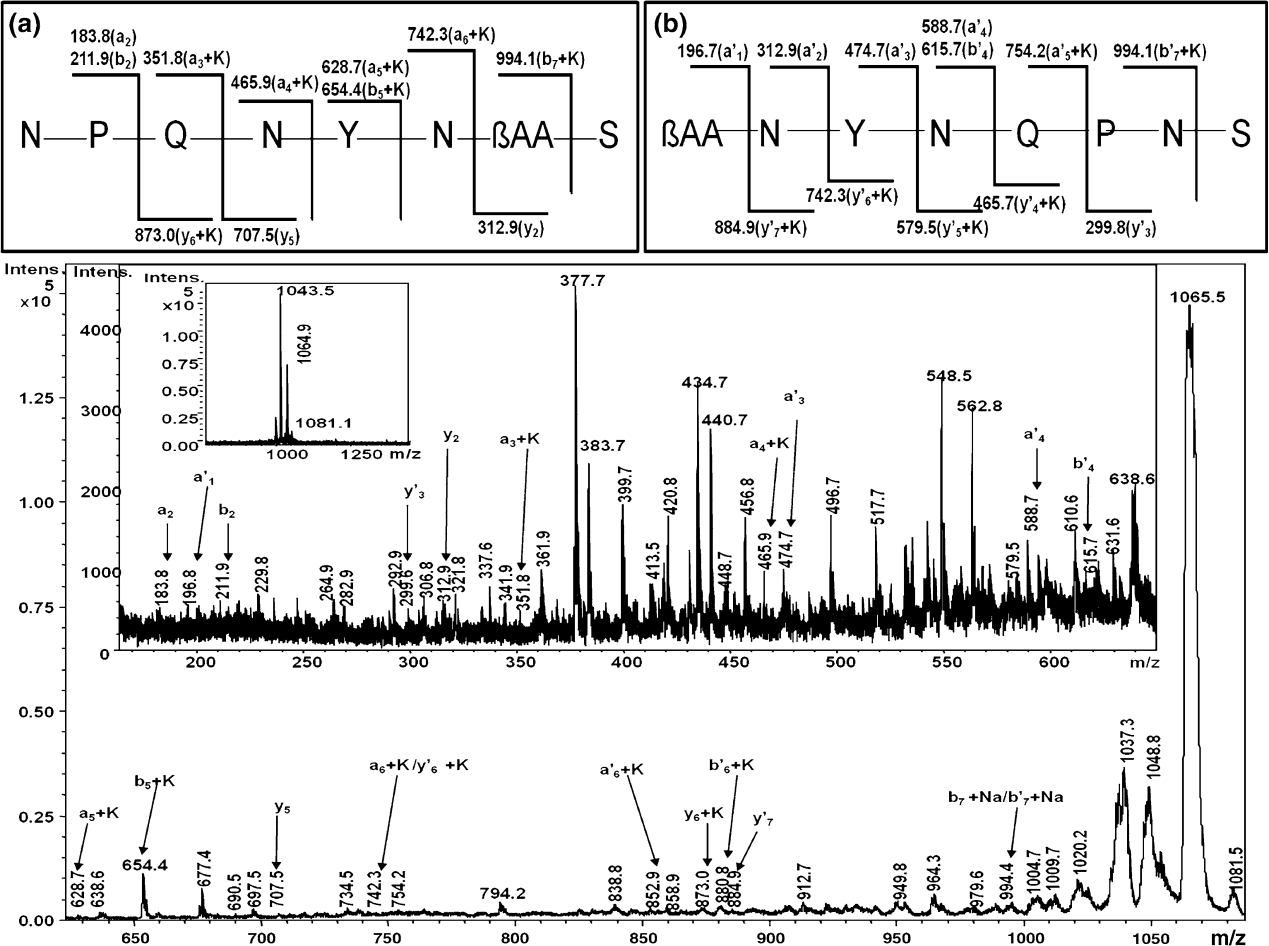
MALDI-MS/MS spectrum of [M+K]^+^ ion at *m/z* 1,081.5 from HPLC fraction P3

In MALDI-MS spectrum of metabolites eluted in HPLC peaks no. P3, the ion at *m/z* 1,081.5 was assigned as [M+K]^+^ ion of iturin A. To confirm its identity, tandem mass spectrometric analysis was carried out. Figure 4 shows the MS/MS analysis of *m/z* 1,081.5 with *a*-, *a′*-, *b*-, *b′*-, *y*- and *y′*-fragment ions. Along with *b*- and *y*-ions some internal ions were also observed. The ions at *m/z* 337.6, 464.9, 562.8, 655.4, 677.4, 838.8, 1,046.2 and 1,064.1 could be assigned as [S-N-P+K]^+^, [S-N-P-Q+K]^+^, [βAA-S-N-P+K]^+^, [N-P-Q-N-Y+K]^+^, [N-βAA-S-N-P+K]^+^, [Y-N-βAA-S-N-P+K]^+^, [M+K-CONH_2_]^+^ and [M+K-NH_3_]^+^, respectively. On the basis of the fragmentation pattern observed, two ring cleavage sites: (1) between Asn^6^-Ser^7^ and (2) βAA-Ser^7^ were predicted. The sequences derived from the fragment ion series corresponding to the predicted ring cleavages were N-P-Q-N-Y-N-βAA-S and βAA-N-Y-N-Q-P-N-S, respectively (Fig. 4).

The fragmentation pattern observed for sodium and potassium adducts of iturin A were different from the fragmentation pattern of protonated isoforms of iturin A. The major product ions from sodium adducts of iturin A were *a*-, *b*- and *y*-ions. This pattern of product ions from sodium adducts has also been reported by others (Papayannopoulos 1995; Rutenbach et al. 2001; Sabareesh et al. 2007). The fragment ions containing Glu^4^ were detected as sodium or potassium adducts while the metal adduct form of fragment ions lacking Glu4 was not observed in respective MS/MS spectrum of sodium ion adducts (*m/z* 1,051.5, 1,065.6, 1,079.5 and 1,093.5) and potassium adducts (*m/z* 1,067.5, 1,081.5, 1,095.5 and 1,109.5), suggesting that possible site of metal interaction was Glu^4^ in iturin A homologues (Papayannopoulos 1995; Sabareesh et al. 2007). The fragmentation pattern showed no cleavage at highly labile Glu^4^-Pro^5^ peptide bond in the sodiated and potassiated iturins. It has been reported that the complexation of iturin homologues with sodium/potassium ions confers the extra-stability to the otherwise highly susceptible bond (X-Pro bond) (Rutenbach et al. 2001). In our studies employing MALDI-TOF MS/MS, only mono-sodium/mono-potassium adducts of iturin A homologues could be detected. Rutenbach et al. (2001) also reported the predominant occurrence of mono sodium/potassium adducts of cyclic iturin A2, synthetic iturin A2 and its shorter analogues. They also proposed that the site for binding of alkali metal ion was present in one of the two β-turns on the interior of the peptide ring of natural iturin A molecule, with C=O oxygen as the chelating atom. The interaction of metal ions in the interior region of peptide ring may play a significant role in the formation of anion selective pores in the target cell membranes.

The isoforms of iturins exists which have same mass but differ in their peptide sequence for example iturin A(C-16) and mycosubtilin (C-16) differ in peptide sequence but have same mass (1,070 Da) (Isogai et al. 1982; Gong et al. 2006). The tandem mass spectrometry can be employed to easily identify such iturin isoforms, as each will yield a unique fragmentation finger print.

### Identification and characterization of surfactin

**Fig. 5:**
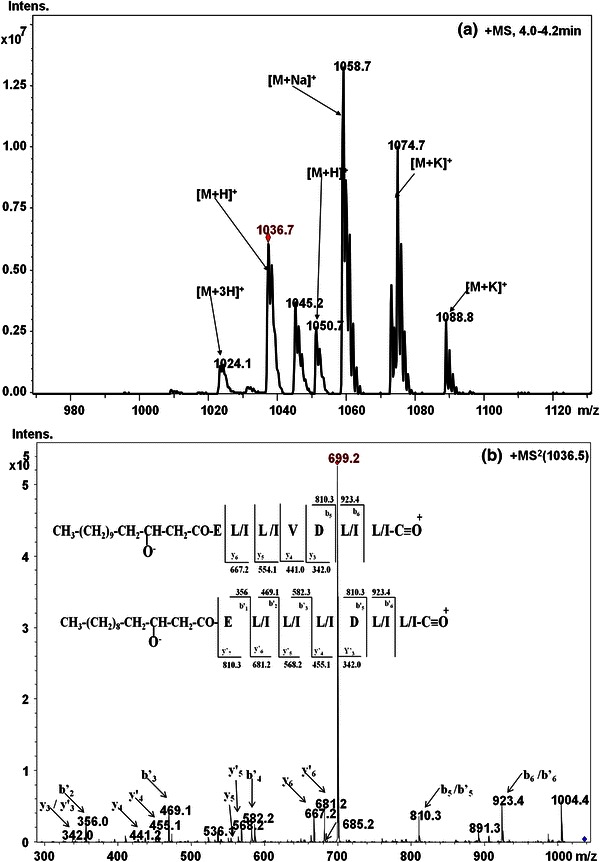
**a** LC-ESI-MS spectrum of surfactin cluster separated at retention time period of 4.0–4.2 min, **b** LC-ESI-MS/MS spectrum of [M+H]^+^ ion at *m/z* 1,036.5

The surfactin group of lipopeptides within the mass range *m/z* 994–1,065 eluted at long retention times from C18 reverse phase HPLC columns and were largely concentrated in the peaks P20–P23. Figure 5a shows the LC-ESI-MS spectrum of surfactins eluted with retention time of 4.0–4.2 min. In the LC-ESI-MS/MS spectrum of ion at *m/z* 1,036.7, series of *b*- and *y*-ions could be easily assigned (Fig. 5b), which resulted upon initial cleavage of protonated ester bond. The previous studies on cyclodepsipeptides have established the facile cleavage of protonated species at ester linkages (Sabareesh et al. 2007). The two peptide sequences of a molecular mass ion at *m/z* 1,036.7 could be deduced based on the fragmentation profile as: β-OH FA-Glu^1^-Leu/Ile^2^-Leu/Ile^3^-Val^4^-Asp^5^-Leu/Ile^6^-Leu/Ile^7^ and β-OH FA-Glu^1^-Leu/Ile^2^-Leu/Ile^3^-Leu/Ile^4^-Asp^5^-Leu/Ile^6^-Leu/Ile^7^. The common peaks at *m/z* 699.2 and 685.2 observed in CID spectrum of the surfactin ion at *m/z* 1,036 could be assigned as an internal protonated fragment ions [(H)Leu/Ile^2^-Leu/Ile^3^-Leu/Ile^4^-Asp^5^-Leu/Ile^6^-Leu/Ile^7^(OH)+H]^+^ and [(H)Leu/Ile^2^-Leu/Ile^3^-Val^4^-Asp^5^-Leu/Ile^6^-Leu/Ile^7^(OH)+H]^+^ with a gain of 18 Da, which is in agreement with observations reported by Hue et al. (2001). The presence of fragment ions with a gain of 18 Da could be due to cleavage of the peptide bond under gas phase upon double hydrogen transfer (Hue et al. 2001; Williams and Brodbelt 2004). The presence of *m/z* 685.2 and 699.2 fragment ions is ubiquitous in CID spectra of all surfactin [M+H]^+^ homologues, thus this fragment ions represent characteristic marker ion for identification of surfactins. All these results were sufficient to establish the sequence identity of protonated surfactin homologues varying in their β-OH fatty acid chain lengths as well as exhibiting variation of Val to Leu/Ile at position 4 in cyclic depsipeptide ring.

**Fig. 6:**
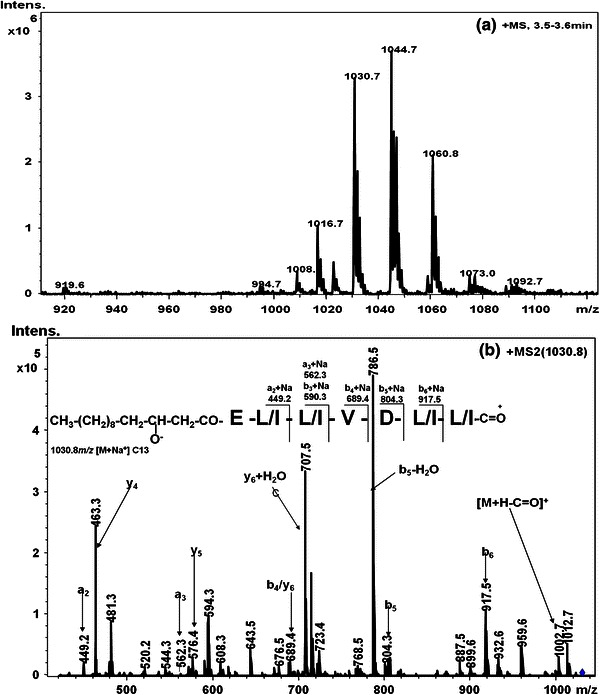
**a** LC-ESI-MS spectrum of surfactin cluster separated at retention time period of 3.5–3.6 min, **b** LC-ESI-MS/MS spectrum of [M+Na]^+^ ion at *m/z* 1,030.8

**Fig. 7:**
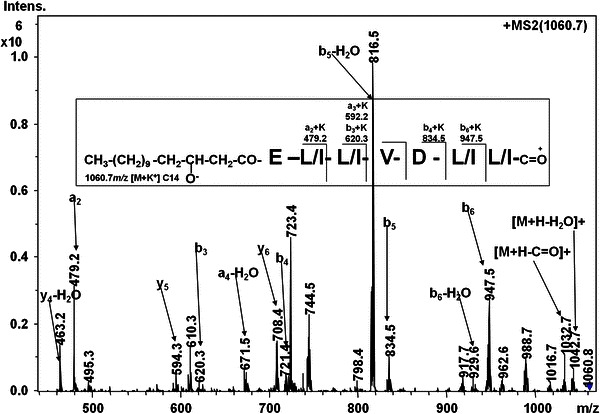
LC-ESI-MS/MS spectrum of [M+K]^+^ ion at *m/z* 1,060.7

The alkali metal ion adducts have been a common feature in the mass spectra of surfactins appearing as minor peaks along with their corresponding protonated precursor ions. The alkali metal ions bind to free carboxylic acid group in surfactin and form metal ion adducts. The sodium and potassium adducts of surfactin have been observed most frequently as these metal ions are ubiquitously present in nature (Hue et al. 2001; Vater et al. 2002). The additions of chloride salts of metal ions to these molecules greatly enhance the relative intensity of corresponding metal ion species in the mass spectra (Eckart 1994; Hue et al. 2001). The precursor ions *m/z* 1,030.8, 1,044.7 and 1,073.0 (Fig. 6a) were assigned as the sodium ion adducts of surfactin homologues with 1,008.7, 1,022.7 and 1,050.7 Da mass, respectively, whereas the precursor ion at *m/z* 1,060.8 (Fig. 7) was assigned as potassium adduct of surfactin homologue with mass of 1,022.7 Da (Hue et al. 2001; Vater et al. 2002; Williams and Brodbelt 2004).

The *a*-, *b*- and *y*-series of fragments ions could be assigned in the CID spectrum of *m/z* 1,030.8 (Fig. 6b; Table 2). The peak at *m/z* 481.3 observed in spectrum of *m/z* 1,030.8 could be assigned as an internal fragment ion [Val^4^-Asp^5^-Leu^6^-Leu^7^(OH)+H+Na]^+^, which is equivalent to *y*_*4*_ + H_2_O. The common peaks at *m/z* 707.5 and 594.3 were observed in this sodium adduct which could be assigned as internal peptide fragment ions [Leu^2^-Leu^3^-Val^4^-Asp^5^-Leu^6^-Leu^7^(OH)+H+Na]^+^ and [Leu^3^-Val^4^-Asp^5^-Leu^6^-Leu^7^(OH)+H+Na]^+^, respectively, which are equivalent to y_6_ and y_5_-ions with addition of water molecule. The presence of these intense fragment ions in ESI-MS/MS spectra of surfactin is a result of cleavage of peptide bond after double hydrogen transfer (Hue et al. 2001).

**Table 2:** Surfactins secreted by *B. subtilis* K1

Maas (*m/z*)	Peptide sequence	Identification of surfactins	References
1,008.6	Cyclo(β-OHFA-E-L/I-L/I-V-D-L/I)	(C_13_) Surfactin (V^4^), [M+H]^+^	This work (Park et al. 1995)
1,030.6	Cyclo(β-OHFA-E-L/I-L/I-V-D-L/I)	(C_13_) Surfactin (V^4^), [M+Na]^+^	
1,022.5	Cyclo(β-OHFA-E-L/I-L/I-V-D-L/I)	(C_14_) Surfactin (V^4^), [M+H]^+^	This work (Park et al. 1995)
1,022.5	Cyclo(β-OHFA-E-L/I-L/I-L/I-D-L/I)	(C_13_) Surfactin (I/L^4^), [M+H]^+^	This work (Kowall et al. 1998)
1,044.5	Cyclo(β-OHFA-E-L/I-L/I-V-D-L/I)	(C_14_) Surfactin (V^4^), [M+Na]^+^	This work (Park et al. 1995)
1,060.6	Cyclo(β-OHFA-E-L/I-L/I-V-D-L/I)	(C_14_) Surfactin (V^4^), [M+K]^+^	This work (Vater et al. 2002)
1,036.5	Cyclo(β-OHFA-E-L/I-L/I-V-D-L/I)	(C_15_) Surfactin (V^4^), [M+H]^+^	This work (Vater et al. 2002)
1,036.5	Cyclo(β-OHFA-E-L/I-L/I-L/I-D-L/I)	(C_14_) Surfactin (I/L^4^), [M+H]^+^	
1,050.6	Cyclo(β-OHFA-E-L/I-L/I-V-D-L/I)	(C_16_) Surfactin (V^4^), [M+H]^+^	This work
1,073.6	Cyclo(β-OHFA-E-L/I-L/I-V-D-L/I)	(C_16_) Surfactin (V^4^), [M+Na]^+^	
1,065.6	Cyclo(β-OHFA-E-L/I-L/I-V-D-L/I)	(C_17_) Surfactin (V^4^), [M+H]^+^	This work

*β-OHFA* β-hydroxy fatty acid

The CID analysis of 1,060.7 showed *a*- and *b*-type of fragment ions (Fig. 7). The fragment ions at *m/z* 723.4 and 610.3 could be assigned as internal ions [Leu^2^-Leu^3^-Val^4^-Asp^5^-Leu^6^-Leu^7^(OH)+H+K]^+^ and [Leu^3^-Val^4^-Asp^5^-Leu^6^-Leu^7^(OH)+H+K]^+^, respectively_._ The ion at *m/z* 495.3 could be assigned as [Val^4^-Asp^5^-Leu^6^-Leu^7^(OH)+H+K]^+^ (Fig. 7). The sequence of sodium and potassium adducts of surfactin homologues revealed Leu at seventh position. The sodium adducts of surfactin (*m/z* 1,030.8, 1,044.7 and 1,073.0) were assigned as homologues with 13-, 14- and 16-C β-OH FA, respectively while potassium adduct *m/z* 1,060.7 was assigned as surfactin homologue with 14-C β-OH FA. The heterogeneity in the Val/Leu/Ile at positions 2, 4 and 7 in the peptide sequence of surfactins is well established (Kowall et al. 1998; Hue et al. 2001; Vater et al. 2002; Williams and Brodbelt 2004). However, differentiation of Leu and Ile from each other is not an easy task using LC–ESI–MS/MS. Hue et al. (2001) employed LSI–MS technique to differentiate between Leu and Ile based upon detection of *d*- and *w*-ions generated upon side chain fragmentation. The *d*- and *w*-ions could not be assigned in the MS_2_ spectra of surfactin homologues and thus, it was not possible to specify whether these homologues contained Leu/Ile at positions 2 and 7 in the peptide sequence.

The protonated and alkali metal ion species of surfactin isoforms characterized from the culture supernatants of *B. subtilis* K1 are summarized in Table 2 along with the peptide variants of surfactins reported by various researchers. The surfactin homologues produced by banyan endophyte, *B. subtilis* K1, comprise C_13_ to C_17_ isoforms which is in agreement to reports for other surfactin producers (Kowall et al. 1998; Hue et al. 2001; Vater et al. 2002; Kim et al. 2010; Williams and Brodbelt 2004).

### Biological activity of lipopeptides

The minimal inhibitory concentrations (MIC) for purified iturin A homologues and crude antifungal extract were determined using double dilution method in 96-well microtitre plate. Table 3 represents MIC for purified iturin fractions and crude antifungal extract. The surfactins could not be purified as some fengycins co-eluted along with surfactins. The MIC values of iturin A1 and A2/A4/A5 against *Candida albicans* were found to be 5 and 10 μg/mL, respectively, which is in agreement to observations reported by Winkelmann et al. (1983). The iturins were found to be more potent against *A. niger* 40211, *C. indicum*, *Alt. brunsii*, and *Clad. herabarum* 1126, in comparison to *Can. albicans*, *Trichosporon* 1110 and *F. oxysporum*. Klich et al. (1991) also reported requirement of higher concentration iturin A to inhibit the growth of *A. parasiticus*, *A. flavus* and *Fusarium moliniforme*. The MIC of crude extract was found to be lower in comparison to both purified iturins. The lipopeptide crude extract is composed of iturin A, iturin C, iturin D, fengycin A1/A1′, fengycin A2/A2′, fengycin B1/B1′, fengycin B2/B2′ and fengycin C1/C1′ as well as surfactins, suggesting synergism amongst these lipopeptides towards fungal antagonism. However, it is not possible to make any conclusive comment on synergism between the lipopeptides produced by *B. subtilis* K1, unless each of them is purified in sufficient concentration and systematic study in this regards is conducted. We have not been able to separate all isoforms of these lipopeptides even after repeated cycles of purification by HPLC as well as attempts using other methods. The main reason for inability to purify them is the extent of heterogeneity amongst these lipopeptides both at the level of amino acid sequence as well as in the length of fatty acid or βAA. Nevertheless, the synergistic action of iturin and fengycin, iturin and surfactin as well as surfactin and fengycin has been documented earlier (Maget-Dana and Peypoux 1994; Ongena et al. 2005; Romero et al. 2007).

**Table 3:** Minimum inhibitory concentration (MIC) of lipopeptides fractions and crude extract of *B. subtilis* K1

Test culture	MIC of lipopeptides containing fractions (μg/mL)		
	Iturin A2	Iturin A3/A4/A5	Crude extract
*A. niger* 40211	2.5 ± 0.03	1.25 ± 0.01	0.63 ± 0.008
*A. paraciticus* 40211	5.0 ± 0.05	5.0 ± 0.05	1.25 ± 0.05
*C. indicum*	5.0 ± 0.05	2.5 ± 0.05	0.063 ± 0.001
*F. oxysporum* 1072	5.0 ± 0.03	5.0 ± 0.04	2.5 ± 0.02
*Alt. brunsii*	2.5 ± 0.03	1.25 ± 0.01	0.063 ± 0.001
*Ca. albicans*	5.0 ± 0.05	10.0 ± 0.08	2.5 ± 0.02
*Trichosporon* sp. 1110	5.0 ± 0.06	10.0 ± 0.07	2.5 ± 0.02
*Clad. herbarum* 1126	5.0 ± 0.03	2.5 ± 0.02	0.031 ± 0.0015

## Conclusion

The paper describes antifungal activity and in detail mass spectrometric investigation of cyclic lipopeptides such as C_13_–C_16_ β-amino fatty acid variants of iturin A as well as iturin C and C_13_–C_17_ β-OH fatty acid isoforms of surfactin exhibiting variation in Val/Ile/Leu at position 4. Seven different variants of iturins as well as of surfactins produced by *B. subtilis* K1 could be identified by tandem mass spectrometry. The MIC of crude extract was lower than purified iturin fractions, suggesting synergism amongst cyclic lipopeptides co-produced by *B. subtilis* K1 in inhibiting the growth of fungi. Further, it would be worth exploring the role of these isoforms in biology of producing bacilli strain.

## Electronic supplementary material

Below is the link to the electronic supplementary material.

Supplementary material 1 (DOCX 613 kb)
