# Supplementary material for: Identification of surfactins and iturins produced by potent fungal antagonist, Bacillus subtilis K1 isolated from aerial roots of banyan (Ficus benghalensis) tree using mass spectrometry
Source: 3 Biotech. 2013 Jul 4;4(3):283–95. doi: 10.1007/s13205-013-0151-3 (PMC4026446; doi:10.1007/s13205-013-0151-3)
Supplement: Supplementary file 1 — Supplementary material 1 (DOCX 613 kb) [file 13205_2013_151_MOESM1_ESM.docx]

**
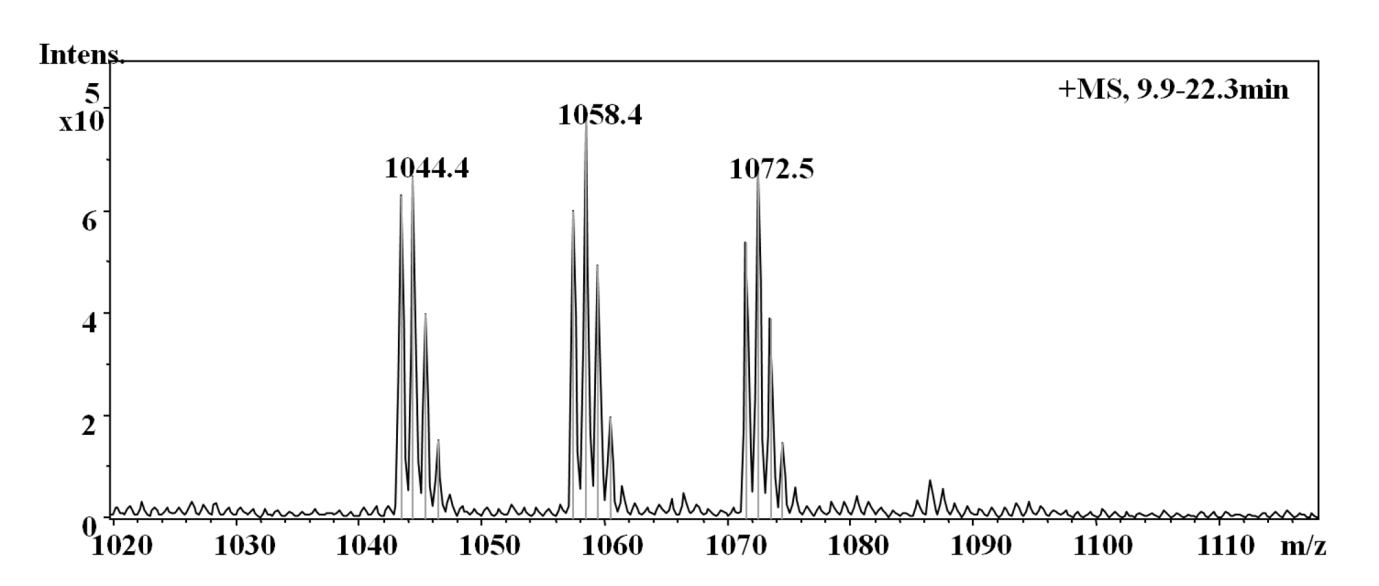
**

**Fig. S1. LC-ESI-MS of spectrum of iturin cluster eluted within the time interval of 9.9 to 22.3 min.**

**
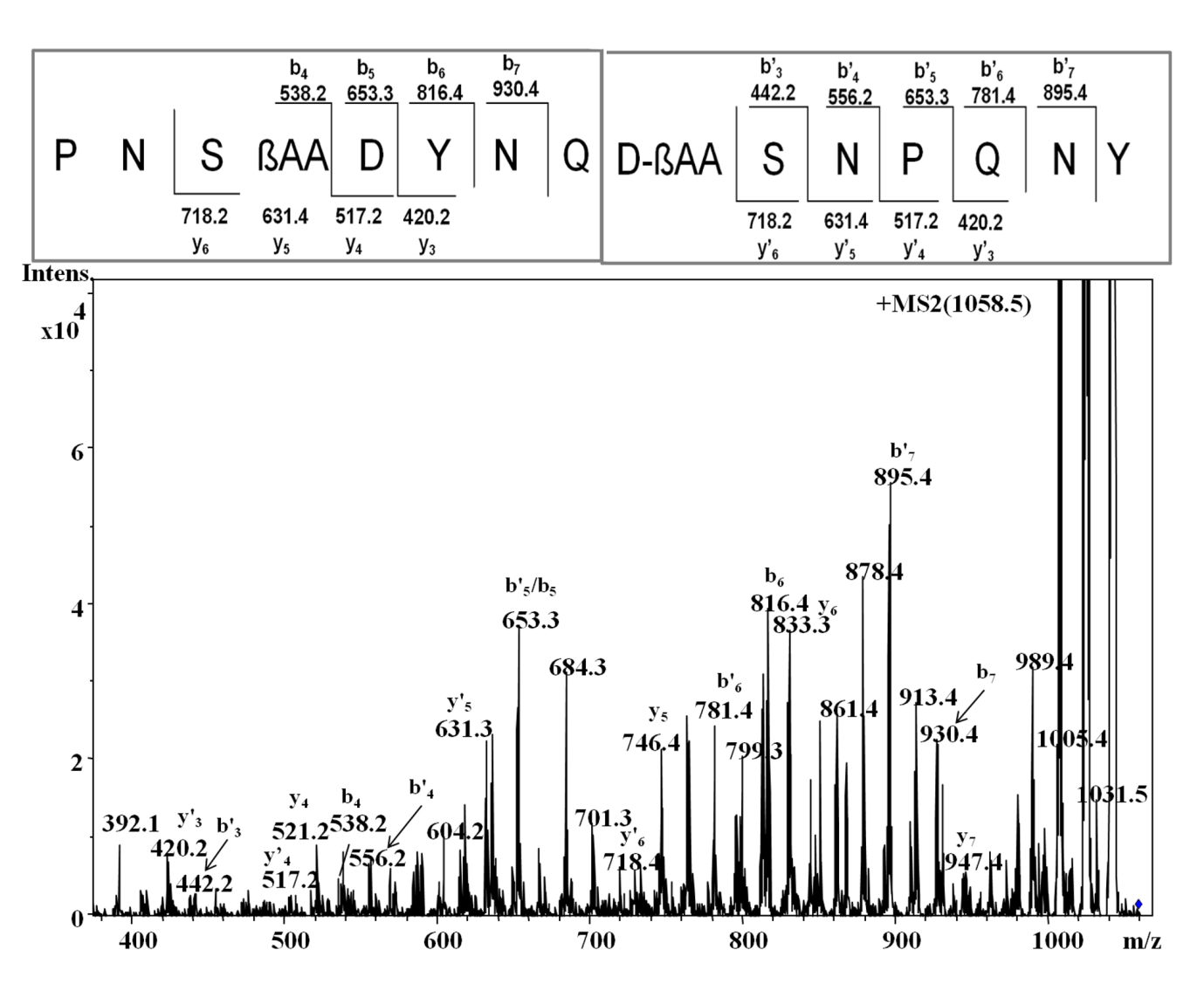
**

**Fig. S2. LC-ESI-MS/MS of spectrum of [M+H]^+^ ion at *m/z* 1058.5.**
